# Gender-Inclusive Profiling of Gamers: Insights into Women’s Emotional and Motivational Pathways

**DOI:** 10.1192/j.eurpsy.2026.10891

**Published:** 2026-07-31

**Authors:** C. M. Castro, D. Neto

**Affiliations:** 1 Psychology, ISPA; 2 Research Center, APPSYCI, Lisbon, Portugal

## Abstract

**Introduction:**

**Background:** Video gaming is a global phenomenon increasingly recognized in mental health research, with potential for both adaptive and maladaptive outcomes. While gaming disorder (GD) has been integrated into the ICD-11, the psychological heterogeneity of players remains understudied, particularly in women and non-binary gamers and across diverse game genres.

**Objectives:**

**Aims** This study investigates the association of mental health, relational contexts, and gaming characteristics with psychological profiles of gamers, focusing on emotional regulation and motivations for play. A central contribution of this study is its inclusivity: it integrates women and non-binary participants and considers all types of video games, addressing gaps in existing literature often limited to male samples or specific genres.

**Methods:**

An international sample of 5,255 gamers (49.9% men, 43.2% women, 9.3% non-binary; aged 16–69, M = 25.6, SD = 6.46) from 112 countries completed validated measures of emotion regulation (DERS-SF), gaming motivation (GMI), mental health (CORE-SFB), attachment (ECR-RS), social interactions, and gaming habits. Latent profile analysis identified four psychological profiles, followed by multinomial logistic regressions to assess associations with mental health, social-relational, and gaming-related covariates.

**Results:**

Four profiles were identified: avoidant (older players, low distress, secure attachment, offline socialization), engaged (largest group, secure attachment, active social integration of gaming), relational (insecure attachment, emotional regulation difficulties, selective online engagement), and dysregulated (younger players, high distress, substance use, online preference). Dysregulated and relational profiles were linked to insecure attachment and mental health problems, while engaged and avoidant profiles reflected secure attachment and adaptive functioning.

**Conclusions:**

These findings highlight the complex interplay of psychological, social, and gaming-related factors shaping player experiences. By including women, non-binary players, and all types of video games, this study provides one of the most inclusive examinations of gaming profiles to date, underscoring the need for gender-sensitive and context-aware prevention and intervention strategies in mental health.

**Disclosure of Interest:**

None Declared

